# Polydopamine‐Modified Black Phosphorous Nanocapsule with Enhanced Stability and Photothermal Performance for Tumor Multimodal Treatments

**DOI:** 10.1002/advs.201800510

**Published:** 2018-08-16

**Authors:** Xiaowei Zeng, Miaomiao Luo, Gan Liu, Xusheng Wang, Wei Tao, Yaoxin Lin, Xiaoyuan Ji, Lin Nie, Lin Mei

**Affiliations:** ^1^ School of Pharmaceutical Sciences (Shenzhen) Sun Yat‐sen University Guangzhou 510275 China; ^2^ Brigham and Women's Hospital Harvard Medical School Boston MA 02115 USA

**Keywords:** black phosphorus nanosheets, enhanced stability, multimodal treatments, polydopamine

## Abstract

As a novel 2D material, black phosphorus (BP) nanosheets are considered as a promising candidate for drug delivery platform for synergistic chemo/photothermal therapy. However, the intrinsic instability of bare BP poses a challenge in its biomedical applications. To date, some strategies have been employed to prevent BP from rapid ambient degradation. Unfortunately, most of these strategies are not suitable for the drug delivery systems. Here, a simple polydopamine modification method is developed to enhance the stability and photothermal performance of bare BP nanosheets. Then, this nanocapsule is used as a multifunctional codelivery system for the targeted chemo, gene, and photothermal therapy against multidrug‐resistant cancer. The enhanced tumor therapy effect is demonstrated by both in vitro and in vivo studies.

Black phosphorus (BP), a novel 2D material, has presently attracted enormous attention in the worldwide.^1^ BP nanosheets (NSs) with different thickness can be produced from bulk BP by exfoliation techniques.^2^ As the most stable allotrope compared to other phosphorus elements,^3^ BP exhibits many unique properties. Unlike graphene, which is a zero bandgap semiconductor,^4^ BP shows a layer‐dependent bandgap that spans from 0.3 eV (a bulk value) to ≈2.0 eV (a monolayer value),^5^ leading to a broad absorption across the ultraviolet and infrared regions.[[qv: 4b,6]] Moreover, due to a large NIR extinction coefficient and high photothermal conversion efficiency, BP is a promising nanomaterial for photothermal therapy (PTT).^6^, ^7^ The final degradation products of BP are phosphate and phosphonate, both of which are nontoxic.^8^ Therefore, BP NSs with good biocompatibility and excellent photothermal performance are suitable for biomedical applications.

Furthermore, compared with other 2D materials such as graphene and MoS_2_, BP NSs possess much higher specific surface area, which could enable more efficient loading of drug. Recently, a few studies have indicated the potential of BP NSs in drug delivery platform.[[qv: 7b,9]] By loading anticancer drug (doxorubicin, DOX) onto the surface of BP sheets, they presented a multifunctional drug delivery system for synergistic photothermal/chemotherapy of cancer. On this basis, we hoped to fabricate a novel multifunctional codelivery platform for combined gene/photothermal/chemotherapy against multi‐drug resistant cancer by loading anticancer drug and siRNA on the same BP NS.

However, it has been revealed that BP is very reactive to oxygen and water,[[qv: 4b,10]] resulting in compositional and physical changes of BP.^11^ The lack of water‐ and air‐stability under ambient conditions hinders its potential biomedical applications. Very recently, some efforts have been made to improve the stability of BP NSs, including ligand surface coordination,^10^, ^12^ covalent aryl diazonium functionalization,[[qv: 11b]] and capping layer protection.[[qv: 11c]] However, most of these strategies are not suitable for the drug delivery systems. These strategies either increased its toxicity or reduced its photothermal performance. The fabrication of high‐performance BP‐based delivery platform is still a challenge due to the difficulty to simultaneously achieve good stability and high photothermal performance.

Mussels, a kind of bivalvia mollusca, which have been presented to immobilize themself closely to all types of substrates surface with high binding strength, even though the surface is wet. The mussels' adhesive capacity may be attributed to the interface between the substrate surface and Mytilus edulis foot protein (Mefp). More and more investigations discovered that the Mefp abound various catecholic amino acid, including the dopamine (DA) and its derivative, which in favor of the high bonding or crosslinking ability of adhesives.^13^ Herein, a simple polydopamine (PDA) modification strategy, which is mussel‐inspired biomimetics, was established to enhance the stability and improve the photothermal performance of the BP NSs‐based codelivery platform. PDA, produced by the oxidative polymerization of DA under weakly alkaline conditions, has been employed as a promising coating in the biological field due to their excellent biocompatibility, biodegradation, and pH response at low pH values.^13^ PDA coating can form extraordinary adhesion on nearly any substrate surfaces and further couple secondary biopolymers with amines/thiols via Michael addition or Schiff base reactions.^14^ Besides, PDA also has strong NIR absorbility and high photothermal conversion efficiency.[[qv: 13b,15]] Therefore, the PDA coating could not only isolates the interior BP NS from oxygen and water to improve its stability but also enhance its photothermal performance.

To improve therapeutic efficacy and safety, it is crucial to increase the site‐specific delivery ability of systemically administered nanoparticles.^16^ As a single‐stranded oligonucleotide, aptamers (Apts) have emerged as a promising targeting agent for specific penetration into biological compartments with non‐immunogenicity.^17^ AS1411 Apt is one of the DNA Apts which can bind to nucleolin (NCL) with high specificity and affinity.^18^ NCL, owing to its selective overexpression on the plasma membrane of a variety of cancer cells (e.g., breast cancer, cervical cancer, liver cancer, and so on), has been widely used as a tumor marker.^18^, ^19^ Herein, to improve its active tumor targeting capacity, the as‐fabricated BP‐R‐D@PDA was further decorated with NH_2_‐PEG‐Apt on the PDA film (designated as BP‐R‐D@PDA‐PEG‐Apt).

In this work, we reported a novel multifunctional co‐delivery system based on BP NSs for targeted gene/chemo/photothermal therapy against multidrug‐resistant cancer. DOX and P‐gp siRNA were employed as model therapeutic substances. P‐gp siRNA can downregulate permeability glycoprotein (P‐gp) expressions on the cancer cell membrane, which is responsible for the P‐gp mediated multidrug resistance (MDR).^20^ The drug‐loaded BP NSs were wrapped in a pH‐sensitive PDA film to improve its stability and photothermal performance. The introduction of Apt on the surface of PDA endowed this co‐delivery system with excellent active tumor targeting ability.

The synthetic process of BP‐R‐D@PDA‐PEG‐Apt was exhibited in **Figure** **1**. The BP NSs used in this work were prepared by a modified liquid exfoliation technique from bulk BP. P‐gp siRNA was adsorbed into the surface of BP in 66.7% ethanol solution and the adsorbed amount of siRNA was ≈4.62 nmol mg^−1^ (Table S1, Supporting Information). Then DOX was loaded via electrostatic adsorption with loading content (LC) of 8.2% (Table S2, Supporting Information). Thereafter, to enhance the ambient stability and phtotothermal performance of bare BP, PDA was modified on the surface of BP NSs in a weakly alkaline condition. Finally, the NH_2_‐PEG‐Apt was introduced to the surface of PDA by Michael addition reaction (Figure S1, Supporting Information), which contributed to the tumor targeting ability as well as the biocompatibility and physiological stability.

**Figure 1:**
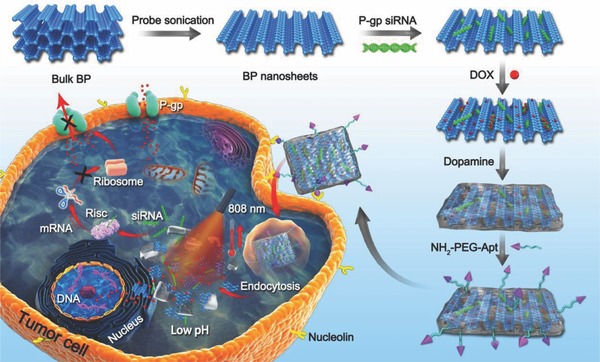
Schematic illustration of the procedure used to fabricate nanostructures and the combined chemo/gene/photothermal targeted therapy of tumor cells.

Transmission electron microscopy (TEM) and atomic force microscopy (AFM) were employed to characterize the morphology of BP nanosheets. As shown in TEM images (**Figure** **2**A–C), the lateral size of bare BP and modified BP NSs were about 200–300 nm, which corresponded to that measured from dynamic light scattering analysis (Figure S3, Supporting Information). After loading P‐gp siRNA and DOX, an easily identified rough surface could be detected (Figure S2, Supporting Information). As shown in the TEM images of BP@PDA and BP@PDA‐PEG‐Apt, a PDA film could be obviously observed. The AFM images in Figure 2D–I revealed that the thickness of BP, BP@PDA, and BP@PDA‐PEG‐Apt was about 5.3, 10.2, and 12.6 nm, respectively. Zeta potential of different samples was also investigated. As shown in Figure S4 of the Supporting Information, the zeta potential of BP‐R‐D@PDA‐PEG‐Apt was −8.2 mV. A slightly negative charge would be appropriate for cell accessibility and NP dispersibility.^21^

**Figure 2:**
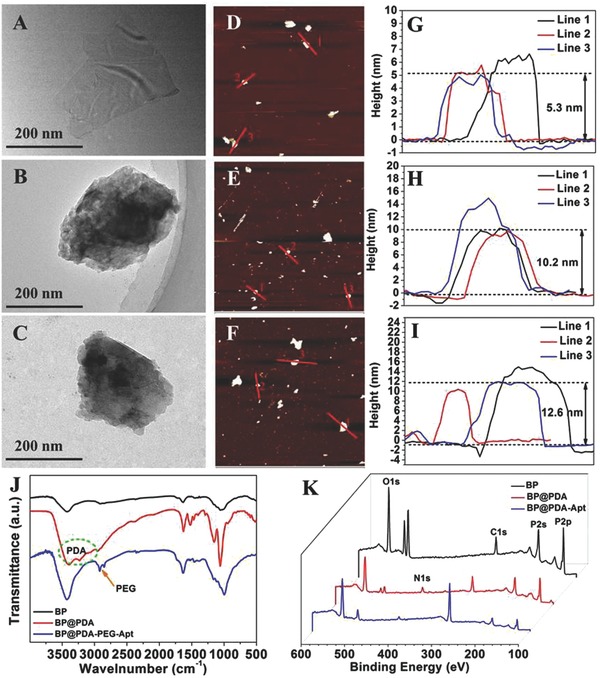
A) TEM image of BP sheets. B) TEM image of BP@PDA. C) TEM image of BP@PDA‐PEG‐Apt. D) AFM image of BP sheets. E) AFM image of BP@PDA. F) AFM image of BP@PDA‐PEG‐Apt. G) Height profiles along the red lines in (D). H) Height profiles along the red lines in (E). I) Height profiles along the red lines in (F). J) FT‐IR spectra of different modified BP sheets. K) XPS spectra of different samples.

Figure 2J shows the Fourier transform infrared spectrum (FT‐IR) of BP NSs, BP@PDA and BP@PDA‐PEG‐Apt. The adsorption peaks at ≈1625 cm^−1^ could be ascribed to P=O stretching mode.[[qv: 7a,22]] After the coating of PDA, a broad and strong band at approximately 3400 cm^−1^ was displayed, which was assigned to the N‐H stretching vibration mode.^23^ The peak at ≈1500 cm^−1^ was due to the benzene ring from PDA. The spectra of the BP@PDA‐PEG‐Apt showed two peaks at approximately 2900 cm^−1^, which was attributed to the C—H and C—H_2_ stretching mode,^24^ indicating the successful modification of the PEG‐Apt.

Raman spectroscopy was performed to characterize the three samples (Figure S5, Supporting Information). For bare BP, three prominent Raman peaks could be observed, which was ascribed to one out‐of‐plane phonon mode A^1^_g_ located at 359.5 cm^−1^ and two in‐plane modes, B_2g_ and A^2^_g_, located at 435.2 and 462.3 cm^−1^, respectively.[[qv: 4b,10,25]] Compared to BP NSs, the A^1^_g_, B_2g_, and A^2^_g_ modes of BP@PDA were blue‐shifted by about 1.8, 1.7, and 3.5 cm^−1^, respectively. When PDA was coated on the surface of BP, the oscillation of P atoms of BP was hindered to some extent, leading to the decrease of corresponding Raman scattering energy. After conjugation of PEG‐Apt, the Raman spectrum displayed another slight blue shift, demonstrating the successful preparation of BP@PDA‐PEG‐Apt.

The chemical composition of the nanosheets was examined by X‐ray photoelectron spectroscopy (XPS) (Figure 2K; Figure S6, Supporting Information). Figure S6B of the Supporting Information shows the N1s spectra of the three samples. The N1s peaks at 400.6 eV were detected from BP@PDA and BP@PDA‐PEG‐Apt, but no N1s peak was observed from the bare BP, confirming the existence of PDA layer. The P2p peak (129.6 eV) intensity of bare BP, BP@PDA and BP@PDA‐PEG‐Apt exhibited a gradual decreasing trend (Figure S6A, Supporting Information). This result was because that there is no P element in the chemical structure of PDA and PEG. Therefore this change indicated the successful modification of the corresponding compounds.

To explore the photothermal property of this co‐delivery platform, the temperature changes was examined upon NIR laser irradiation (808 nm, 10 min). As displayed in **Figure** **3**A, both bare BP and BP‐based therapeutic platform showed a clear temperature elevation. As expected, the photothermal efficiency of BP@PDA (Δ*T* = 27.1 °C) was enhanced as compared to bare BP (Δ*T* = 24.1 °C). The enhanced photothermal response of BP@PDA may be ascribed to the PDA coating, which also has high photothermal conversion efficiency. Moreover, the PDA‐coated BP NSs exhibited both concentration‐dependent and laser‐power‐dependent photothermal property (Figure S7, Supporting Information). As shown in Figure 3B, after irradiation with NIR light for 5 cycles, the process of temperature changes did not significantly change, suggesting satisfactory photostability. Therefore, the PDA coating could improve the photothermal performance of BP NSs, and the high photothermal conversion efficiency as well as excellent photostability made PDA‐modified BP NSs highly promising PTT agents.

**Figure 3:**
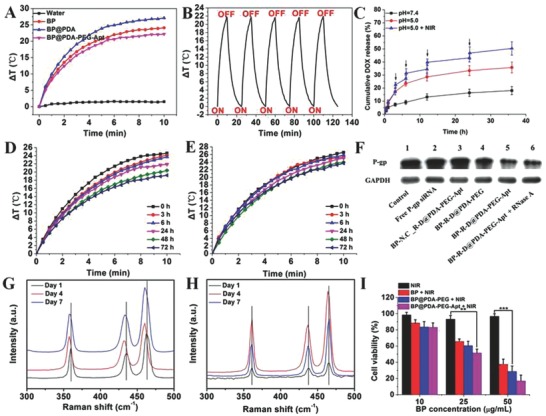
A) Temperature elevation curves of pure water, BP, BP@PDA, and BP@PDA‐PEG‐Apt solution under 808 nm laser irradiation (1.0 W cm^−2^) for 10 min. B) Heating of a suspension of the BP@PDA‐PEG‐Apt in water for five laser on/off cycles with an 808 nm NIR laser at power density of 1.0 W cm^−2^. C) Drug release kinetics of BP‐R‐D@PDA at pH = 7.4 and pH = 5.0 (in the absence or presence of 1.0 W cm^−2^ NIR laser), ↓: NIR irradiation for 0.1 h. D,E) Photothermal heating curves of the BP NSs and BP@PDA, respectively, after storing in water for different periods of time and being irradiated with the 808 nm laser (1 W cm^−2^) for 10 min. F) Western blot analysis. G,H) Raman spectra acquired from the bare BP NSs and BP@PDA, respectively, after storing in water for different days. I) The relative cell viabilities of MCF‐7 cells incubated with various concentrations of BP, BP@PDA‐PEG, and BP@PDA‐PEG‐Apt with NIR laser irradiation (808 nm, 1 W cm^−2^, 10 min).

As shown in Figure 3D,E, in the beginning, the solution temperature of bare BP increased by 24.6 °C after NIR laser irradiation (808 nm, 1.0 W cm^−2^) for 10 min. However, after 72 h, the temperature rise of the BP solution was 19.2 °C, indicating the degradation of photothermal performance. In comparison, BP@PDA exhibited much better photothermal stability. After 3 days, the temperature rise of the BP@PDA solution was 23.7 °C which was close to the initial value of 26.5 °C. The photothermal characteristics of BP and BP@PDA were further examined by Raman spectra. Comparing with obvious red‐shift of the Raman spectra of bare BP after storing in water for 7 days (Figure 3G,H), no significant change was observed from that of the BP@PDA. The hydrodynamic size of BP@PDA NSs in air and water was further monitored over a span of two weeks (Figure S8, Supporting Information). It can be observed that the sizes of these NPs had no significant change in both of these two conditions. These results demonstrated that PDA encapsulation could effectively prevent the degradation of BP and maintain good photothermal stability in water and air.

The controlled and sustained drug release performances of BP‐R‐D@PDA were studied under different pH values (pH 7.4 and 5.0) with or without NIR laser irradiation (Figure 3C). Within 24 h, ≈33.4% of DOX was released from the BP‐R‐D@PDA at pH 5.0 (simulating tumor acidic microenvironment), while the amount of DOX released form BP‐R‐D was 39.2% over the same time period (see Figure S9A, Supporting Information). This result suggested that the PDA “capsule” wrapping out of the drug‐loaded BP NSs could effectively suppress burst drug release. The slowly released anticancer drug would be favorable for the long and continued therapy of tumor and improving the patients' compliance.^26^ As shown in Figure 3C, the DOX releasing rate of BP‐R‐D@PDA at pH 5.0 was much faster than that at pH 7.4 (mimicking the normal physiological microenvironment). The pH‐dependent manner may be due to the pH sensitivity of PDA coating and the protonation of the amino group in DOX molecules.[[qv: 20a,26a]] Under acidic conditions of tumor microenvironment, the external PDA coating was partially peeled off from the BP NSs, thus resulting in a faster drug release. That is to say, the PDA coating formed on the surface of BP acted as a protected capsule, which controlled the DOX release in the tumor. Moreover, the photoresponsible drug release behavior was also studied. After treated with an NIR laser (808 nm, 1.0 W cm^−2^, 6 min for each pulse) at pH 5.0, the temperature of the BP‐R‐D@PDA was gradually increased, which leading to the cumulative DOX release amount was increased significantly, and it reached 46.9% after four times irradiation. In short, the enhanced DOX release behavior upon NIR irradiation could be ascribed to the effective photothermal heating property of PDA coating and BP NSs. We further checked the TEM of BP‐D@PDA after NIR irradiation for 30 min (Figure S10, Supporting Information). Compared with Figure 2B, the PDA layer outside the BP NSs partially broke up and peeled off, indicating the decomposition of the PDA film under NIR irradiation. Besides, a decomposition of internal BP NSs can also be observed. Therefore, it is safe to assume that after irradiation for a certain time, the PDA layer began to decompose, allowing the loaded drug to release. Meanwhile, the BP also gradually decomposed due to the exposure to NIR, which further induced the release of drug.

Moreover, the drug release behavior of BP‐R‐D@PDA‐PEG‐Apt was also investigated (Figure S9B, Supporting Information). It was found that BP‐R‐D@PDA‐PEG‐Apt and BP‐R‐D@PDA had similar drug‐release behaviors, which indicated that the PEG‐Apt modification on the PDA surface did not influence the drug release property. Consequently, the pH‐sensitive and NIR irradiation‐triggered drug release could effectively enhance antitumor efficacy and minimize the side effect of the drug.

As shown in Figure 3F and Figure S11 (Supporting Information), compared to control group, the naked P‐gp siRNA and negative control siRNA hardly influenced the P‐gp expression. BP‐R‐D@PDA‐PEG decreased the P‐gp expression to some extent. Meanwhile, BP‐R‐D@PDA‐PEG‐Apt evidently influenced the expression of P‐gp (≈68% decrease), which was due to the higher cellular uptake of siRNA‐loaded BP NSs after modification of Apt. In order to evaluate the stability of P‐gp siRNA nanocomplexes against nuclease, BP‐R‐D@PDA‐PEG‐Apt was incubated with RNase A (10 µg mL^−1^) for 1 h at room temperature. As can be seen in the picture, the protein expressions of P‐gp in the cells exposed to BP‐R‐D@PDA‐PEG‐Apt with and without RNase A were comparable, indicating that the P‐gp siRNA was well protected by PDA‐coated BP NSs. We further assessed the P‐gp knockdown effect of the NPs in vivo and the results are shown in Figure S12 of the Supporting Information. Integration of all the data demonstrated a lower P‐gp expression in the tumors from the animals treated with P‐gp siRNA‐loaded NPs compared to saline controls. However, no significant reduction of P‐gp expression in the MCF‐7/MDR matrix was observed in the groups treated with free siRNA or NPs containing scrambled siRNA. Therefore, our co‐delivery system could not only give a good protection to siRNA but also effectively suppress the P‐gp expression both in vitro and in vivo.

The photothermal cytotoxicity of different NSs was assessed in MCF‐7 and MCF‐7/ADR cells by the MTT assay. As shown in Figure 3I and Figure S13 (Supporting Information), treatment of NIR laser irradiation alone did not significantly influence cell growth. After laser irradiation, BP presented obviously strong cytotoxicity at the concentration of 25 µg mL^−1^. However, both PDA‐coated BP NSs exhibited stronger photothermal cytotoxicity than bare BP, suggesting that PDA film enhanced the photothermal performance of BP, which corresponded to the in vitro photothermal efficiency assay.

MCF‐7 and MCF‐7/ADR cell lines were used to study the cellular uptake behavior. Cellular uptake results of DOX depicted a time‐dependent manner for both of the two cell lines. For MCF‐7 cells (Figure S14, Supporting Information), the internalization efficiency of free DOX was much higher at both time points compared to other DOX formulations. The higher cellular uptake was perhaps due to the passive diffusion mechanism of free DOX.[[qv: 20a,27]] Negligible green fluorescence (FAM‐siRNA) could be observed in the free DOX group, which indicated that naked siRNA could barely be internalized by cells. After treated with DOX–siRNA‐loaded BP NSs, both strong red (DOX) and green fluorescence intensity were detected in the perinuclear region of the cytoplasm, demonstrating that DOX and siRNA could be effectively codelivered by our nanoplatform.For MCF‐7/ADR cells (**Figure** **4**A; Figure S15, Supporting Information), the DOX fluorescence intensity was very weak in free DOX group. The result was because that P‐gp was overexpressed in MCF‐7/ADR cells and pumped out the intracellular drugs. However, cells treated with DOX and siRNA encapsulated in PDA‐coated BP NSs showed improved accumulation of drugs. The enhanced accumulation could be ascribed to the reduced drug efflux due to the downregulation of P‐gp by carried P‐gp siRNA. The treatment of cells with BP‐R‐D@PDA‐PEG‐Apt showed the highest DOX fluorescence, which was attributed to selective uptake via Apt receptors (NCL). Next, we carried out a receptor competition experiment. Both of the red and green fluorescence intensity significantly decreased after incubation with an excessive amount of free AS1411 aptamers, which implied the important role of Apt in the active tumor targeting. Flow cytometry was also employed to evaluate the cellular uptake behavior and the results were in good agreement with the findings of fluorescence microscope (Figure 4B,C; Figure S16, Supporting Information).

**Figure 4:**
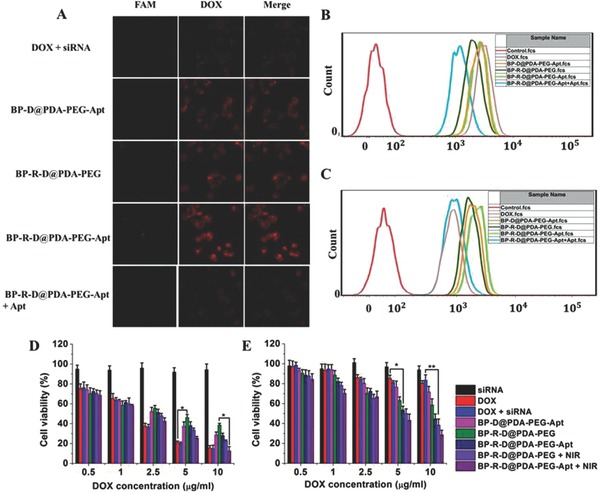
A) Confocal laser scanning microscopy images of MCF‐7/ADR cells after 24 h incubation. Green color: FAM‐labeled siRNA. Red color: DOX. Flow cytometric histogram profiles of DOX in B) MCF‐7 and C) MCF‐7/ADR cells after 24 h incubation. Relative viabilities of tumor cells after different types of treatment for 48 h. D) MCF‐7 cells, and E) MCF‐7/ADR cells (*P< 0.05, **P < 0.01).

In vitro cytotoxicity studies of free DOX, DOX‐loaded nanocarrier, and free nanocarriers were assessed by MTT assay (Figure 4D,E; Figures S17 and S18, Supporting Information). As Figure S18 of the Supporting Information displayed, no substantial cytotoxicity of drug free BP‐based nanocarriers was observed for both MCF‐7 and MCF‐7/ADR cell lines, indicating the good biocompatibility of our codelivery platform. For MCF‐7 cells, free DOX exhibited an overall higher cytotoxicity than other DOX formulations, which could be attributed to that DOX was directly located in nucleus, while the release of DOX from other nanocarriers required a specific period. However, owning to the drug resistance of MCF‐7/ADR cells, the inhibition ratio of cells was less than 20% after exposed to 10 µg mL^−1^ DOX for 48 h. By contrast, DOX–siRNA‐BP NSs led to higher cytotoxicity in MCF‐7/ADR cells than DOX. The increased cytotoxicity was ascribed to the knockdown of P‐gp on the cell membranes, which could abolish the P‐pg‐mediated resistance to apoptosis induced by DOX in MCF‐7/ADR cells. Moreover, it could be observed that cells irradiated with NIR laser showed much lower cell viability than those without irradiation, demonstrating the potential application of the PDA‐coated BP NSs for combined gene/chemo/photothermal therapy.

Four week‐old female sever combined immunodeficient (SCID) mice and healthy male Sprague–Dawley (SD) rats aged 5–6 weeks were used for the animal experiment after purchasing them from the Sun Yat‐sen University Laboratory Animal Center. All the protocols for the proposed in vivo experiments were approved by the Administrative Committee on Animal Research in Sun Yat‐sen University. We further investigated the photothermal efficacy of the BP‐based therapeutic platform. The IR thermal images of mice were shown in **Figure** **5**A. Clearly, the tumor treated with PBS did not show great temperature increase after the laser irradiation. On the contrary, the temperatures of tumors treated with BP@PDA‐PEG and BP@PDA‐PEG‐Apt significantly increased after laser irradiation. The tumor temperature of BP@PDA‐PEG‐Apt group rapidly increased to 54.7 °C within 5 min, which was high enough for effective tumor ablation. The quantitative analysis of tumor temperature change was shown in Figure S19 of the Supporting Information.

**Figure 5:**
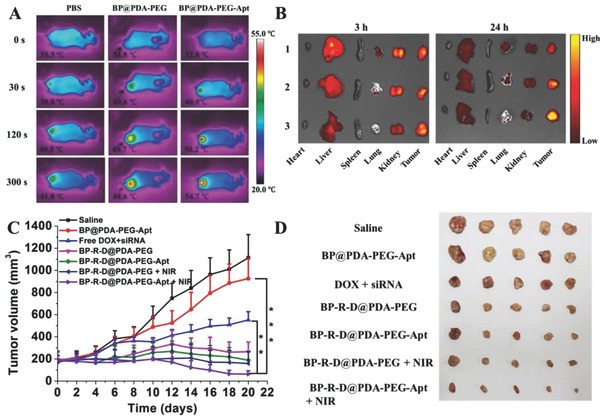
A) In vivo IR thermal images. B) Ex vivo fluorescence images of major organs and tumors after systemically administration at 3 and 24 h. Lane 1, 2, and 3 were represented free DOX, BP‐R‐D@PDA‐PEG, and BP‐R‐D@PDA‐PEG‐Apt, respectively. C) Inhibition of tumor growth after different treatments. D) Morphology of tumors removed from the sacrificed mice in all groups at the end point of study (**P < 0.01, ***P < 0.001).

In vivo biodistribution of the free DOX and DOX‐loaded BP NSs was monitored in MCF‐7/ADR tumor‐bearing nude mice at determined time points after tail vein injection using an in vivo imaging system (Figures 5B and S20, Supporting Information). As shown in the picture, both free DOX and DOX‐loaded BP NSs underwent a rapid distribution in tumor within 3 h. However, a high intensity of DOX in the liver and kidney was also observed. The high concentration of DOX in kidney suggested that renal excretion could be possibly a primary elimination route for DOX.^28^ At 24 h postinjection, only weak DOX fluorescence in tumor could be visualized for free DOX group. By contrast, the fluorescence intensity of both nonoformulations in the tumor tissues was still very strong. This result was ascribed to prolonged systemic circulation in blood resulting from PEGylation and the EPR effect.^29^ Moreover, the DOX fluorescence of BP‐R‐D@PDA‐PEG‐Apt at tumor site was much stronger than that of BP‐R‐D@PDA‐PEG, demonstrating a good tumor targeting ability.

The pharmacokinetic profiles of free DOX and DOX‐loaded BP NSs were studied in Sprague–Dawley (SD) rats (Figure S21, Supporting Information). Free DOX had a rapid clearance from blood with a plasma half‐life (*t*_1/2_) of ≈0.22 h. By contrast, BP‐R‐D@PDA‐PEG presented a longer circulation time with about 14 times prolonged t_1/2_ (≈3.1 h) than the free DOX. The prolonged retention time might be attributed to the long‐circulating effect of PEGylation and relatively larger molecular weight. These results suggested that DOX‐loaded BP NSs had a longer blood circulation time than free DOX, which resulted in a better chance for drug accumulation at tumor sites.

The antitumor efficacy of drug free nanoplatform and different DOX formulations was studied in a subcutaneous xenograft tumor model. During monitoring period, no obvious weight loss was observed (Figure S22A, Supporting Information), indicating that the treatments did not produce serious toxicity and side effect to the tumor bearing mice. As displayed in Figure 5C, DOX + siRNA group showed moderated tumor growth inhibition, suggesting that chemotherapy alone could not effectively kill the tumor cells. Compared to chemotherapy group, the tumor growth was significantly inhibited in the gene and chemo combined treatment groups, which was due to the fact that P‐gp siRNA inhibited the drug resistance to a certain extent. Excitingly, tumors in the BP‐R‐D@PDA‐PEG‐Apt + NIR treatment group showed the best inhibition performance. This was ascribed to the facts that tumor targeting ability led to more accumulation of NSs, and the heat produced by NIR irradiation could effectively kill tumor cells and also improved the release of DOX from BP carriers. The tumor morphology and weight of each group were exhibited in Figure 5D and Figure S22B (Supporting Information), respectively. The results demonstrated that the tumor cells could be killed more effectively under the combined action of gene, chemo, and photothermal therapy.

In vivo toxicity of BP‐based NSs was examined using the hematoxylin and eosin staining test (Figure S23, Supporting Information). The pathological images of major organs (heart, liver, spleen, lung, and kidney) showed no obvious pathologic changes in all groups, validating the biocompatibility of these nanosheets, which is very crucial for in vivo biomedical applications. The tumor from DOX and siRNA‐treated mice was only partially destroyed. However, more tumor necrosis could be found in tumors with treatments of drug nanoformulations. BP‐R‐D@PDA‐PEG‐Apt + NIR group leaded to the most severe destruction of tumor cells, which was due to the targeted combinational gene, chemo, and photothermal therapy.

In summary, we fabricated a multifunctional drug delivery platform based on PDA‐modified BP sheets. This delivery system showed enhanced stability and phtotothermal performance than bare BP. The drug release experiment indicated pH‐responsive and NIR irradiation‐triggered drug release behavior. This codelivery system can effectively transport P‐gp siRNA and DOX into tumor cells and inhibit the drug resistance to some extent. The selective cell targeting ability of our carriers was demonstrated by cellular uptake and cellular targeting assay. Both in vitro and in vivo studies showed an excellent multimodal therapy effect in inhibiting tumor cell proliferation.

## Conflict of Interest

The authors declare no conflict of interest.

## Supporting information

Supplementary
