# Supplementary material for: Polydopamine‐Modified Black Phosphorous Nanocapsule with Enhanced Stability and Photothermal Performance for Tumor Multimodal Treatments
Source: Adv Sci (Weinh). 2018 Aug 16;5(10):1800510. doi: 10.1002/advs.201800510 (PMC6193171; doi:10.1002/advs.201800510)
Supplement: Supplementary file 1 — Supplementary [file ADVS-5-1800510-s001.pdf]

Copyright WILEY-VCH Verlag GmbH & Co. KGaA, 69469 Weinheim, Germany, 2018.

ADVANCED SCIENCE
Open Access

Supporting Information

for *Adv. Sci.,* DOI: 10.1002/advs.201800510

Polydopamine-Modified Black Phosphorous Nanocapsule with Enhanced Stability and Photothermal Performance for Tumor Multimodal Treatments

*Xiaowei Zeng, Miaomiao Luo, Gan Liu, Xusheng Wang, Wei Tao, Yaoxin Lin, Xiaoyuan Ji, Lin Nie, and Lin Mei**

Copyright WILEY-VCH Verlag GmbH & Co. KGaA, 69469 Weinheim, Germany, 2018.

Supporting Information

Polydopamine-Modified Black Phosphorous Nanocapsule with Enhanced Stability and Photothermal Performance for Tumor Multimodal Treatments

*Xiaowei Zeng, Miaomiao Luo, Gan Liu, Xusheng Wang, Wei Tao, Yaoxin Lin, Xiaoyuan Ji, Lin Nie, and Lin Mei**

Dr. X. Zeng, M. Luo, Dr. G. Liu, Dr. X. Wang, Dr. Y. Lin, Dr. X. Ji, L. Nie, Prof. L. Mei

School of Pharmaceutical Sciences (Shenzhen), Sun Yat-sen University, Guangzhou 510275, China

E-mail: meilin7@mail.sysu.edu.cn (L. Mei)

Dr. W. Tao

Brigham and Women's Hospital, Harvard Medical School, Boston, Massachusetts 02115, United States

Materials and methods

Materials

The bulk BP was purchased from Smart-Elements. Dopamine hydrochloride, MTT, 1-Methyl-2-pyrrolidinone (NMP), dimethyl sulfoxide (DMSO), tris-(2-carboxyethyl)-phosphine hydrochloride (TCEP) and methanol were all purchased from Sigma-Aldrich (St. Louis, MO, USA). Methoxy-PEG_{2k}-amine (NH_2-PEG) and maleimide-PEG_{2k}-amine (NH_2-PEG-MAL) were provided by Shanghai Yare Biotech, Inc. (Shanghai, China). Doxorubicin hydrochloride (DOX) was bought from Dalian Meilun Biology Technology Co., Ltd. (Dalian, China). All other chemicals and reagents of the highest quality were commercially available and used as received. Human breast cancer cell line MCF-7 and drug-resistant human breast cancer cell line MCF-7/ADR were from American Type Culture Collection (ATCC, Rockville, MD). Aptamer AS1411 (5'-GGT GGT GGT GGT TGT GGT GGT GGT GGT TTT TTT TTT-thiol-3', Apt-SH) was provided by Sangon Biotech. (Shanghai, China). P-gp siRNA (5'-CGG AAG GCC UAA UGC CGA ATT-3'), scrambled control for P-gp siRNA and fluorescence FAM labeled siRNA (FAM-siRNA) were obtained from GenePharma Co., Ltd. (Suzhou, Jiangsu, China). Anti-Pgp antibody was purchased from Abcam (Cambridge, MA).

Preparation of BP nanosheets

The black phosphorus nanosheets were prepared using a simple modified liquid exfoliation of corresponding bulk sample.[1] In brief, 10 mg of bulk black phosphorus was dispersed in 20 mL of 1-Methyl-2-pyrrolidinone (NMP). The NMP was utilized to reduce the oxidation. The mixture solution was then sonicated in ice bath for 9 h with a sonic tip (Amplifier: 25%, On/Off cycle: 10 s/5 s). The ice water was used to avoid a relatively high temperature of the system. Afterward, the resulting brown suspension was centrifuged at 2000 rpm for 10 min to remove the residual unexfoliated bulk BP particles and the supernatant was carefully collected and stored under 4 °C for further use. Before use, the collected supernatant was centrifuged at 9000 rpm for 5 min to remove NMP.

Preparation of BP-siRNA

Briefly, 1 mg of BP was dispersed in 0.2 mL ethanol in 2 mL centrifuge tube, to which an aqueous solution of Guanidine Hydrochloride (4 M, 50 µL) and siRNA solution (1.5 nmol, dissolved in 50 µL DEPC-treated Milli-Q water) were added. The mixture was continuously shaken at 150 rpm for 0.5 h. The precipitate (BP-R) was collected by centrifugation at 9000 rpm for 5 min. The loading efficiency (LE) and encapsulation efficiency (EE) of siRNA were calculated using a previously published method.[2]

Drug loading

Chemotherapy DOX was loaded on BP NSs under a weakly alkaline condition in ethanol. In brief, 1 mg of BP-R NSs were mixed with 2 mL of DOX ethanol solution (1 mg mL^{-1}), followed by an addition of 8 µL NaOH aqueous solution (20 mg mL^{-1}). After stirred in dark for 30 min, the obtained DOX loaded BP NSs (BP-R-D) were gathered by centrifugation at 9000 rpm for 5 min and washed with water. The DOX loading content (LC) was determined by a previously published method.[3]

PDA encapsulation

1 mg of BP-R-D was suspended in 1 mL ethanol. Then 10 µL dopamine hydrochloride aqueous solution (150 mg mL^{-1}) and 25 µL NaOH aqueous solution (20 mg mL^{-1}) were added. The mixture was allowed to stir in the dark for 2.5 h. The BP-R-D@PDA was obtained by centrifugation at 9000 rpm for 5 min and washed with water.

PEG-Apt conjugation

10 OD of Apt-SH was dissolved in 500 µL tris-HCl buffer（pH=7.4, 10 mM）and then mixed with 1 mg of NH_2-PEG-MAL and 20 µg of TCEP (to prevent oxidation of thiol). The mixture was stirred in the dark for 3 h to obtain NH_2-PEG-Apt. After that, BP-R-D@PDA was added into the above solution and adjusted to pH 8.5 using NaOH solution. After stirring for 1 h, the final product (designated as BP-R-D@PDA-PEG-Apt) was collected by centrifugation (9000 rpm, 5 min) and washed with deionized water.

Characterization of BP-based NSs

Transmission electron microscopy (TEM) images were acquired using FEI Tecnai G2 F30 transmission electron microscope. Atomic force microscopy (AFM) was performed on Bruker Diension Icon microscope. The Fourier transform infrared (FT-IR) experiment was carried out on a Thermo Scientific Nicolet iS 50 spectrometer using KBr method. Raman spectra were obtained on high-resolution confocal Raman microscope (HORIBA LabRAM HR800) equipped with 532 nm laser as the excitation source. X-ray photoelectron spectroscopy (XPS) was carried out using a X-ray photoelectron spectroscopy (Axis HSi, Kratos Ltd., UK) with Al Kα radiation (1486.6 eV photons, 150 W) as the X-ray source for excitation. The size and zeta potential of samples were measured on Malvern Mastersizer 2000 (Zetasizer Nano ZS90, Malvern Instruments Ltd., UK).

Stability evaluation of BP and BP@PDA

To evaluate the influence of PDA encapsulation on BP stability, bare BP and BP@PDA NSs with the same amount of BP concentration (100 μg mL^{-1}) were dispersed in water and exposed to air for 7 days and then their relevant properties were tested at predetermined time intervals.

***In vitro* pH-responsive and NIR-triggered kinetics**

For pH-responsive release, 1 mL of BP-R-D, BP-R-D@PDA, and BP-R-D@PDA-PEG-Apt (5 mg mL^{-1}) was sealed in a dialysis bag (MWCO=3,500, Shanghai Sangon, China), and then merged in 10 mL PBS solutions (pH 7.4 or 5.0). The suspension samples were shaken (120 rpm 37 °C) for 36 h. At certain time intervals, 500 μL of outside release media was collected and the amount of DOX in the solution was measured with an absorbance spectrometer. Then 500 μL of fresh media was added. For NIR-triggered release, the experiments were carried out at pH = 5.0 with an 808 nm NIR laser irradiation (6 min, 1 W cm^{-2}).

Measurement of *in vitro* photothermal property

The photothermal properties of different NSs (bare BP, BP@PDA, BP@PDA-PEG-Apt) were measured by recording the temperature changes of various

solutions with various concentrations of NSs (10−200 μg mL^{-1}) under 808 nm NIR laser irradiation (Shanxi Kaisite Electronic Technology Co., Ltd., Xi'an, China) with laser power density of 1.0 W cm^{-2}. The influence of power density on the photothermal performance was also investigated. BP@PDA-PEG-Apt was irradiated under NIR laser at different power densities (0.5–2.0 W cm^{-2}), and the temperature changes were recorded by an infrared thermal imaging camera (Ti450, Fluke, USA)..

Cell culture and intracellular uptake

The human breast cancer cell line sensitive to doxorubicin (MCF-7) were cultured in Gibco® RPMI 1640's culture medium (Life Technologies Co., Carlsbad, USA) supplemented with 10% (v/v) fetal bovine serum (Gibco), antibiotics penicillin (100 U mL^{-1}) and streptomycin (100 μg mL^{-1}) in a 5% CO_2-humidified atmosphere at 37 °C. The drug resistant human breast cancer cell line (MCF-7/ADR) was maintained in 2 μg mL^{-1} of DOX (made up from 1 mg/mL stock in deionized water) in the above-mentioned culture medium.

Intracellular uptake assay was performed using a fluorescence method. MCF-7 or MCF-7/ADR cells were seeded (1×10^6 cells per dish) in a 20 mm glass-bottom Petri dish for 24 h to reach confluence. The culture medium was replaced with fresh media containing DOX or DOX-loaded BP NSs at the concentration of 5 μg mL^{-1} DOX and cells were incubated for 2 h or 24 h to allow uptake to take place. Then the cells were washed three times with cold PBS, treated with 4% paraformaldehyde solution for 20 min. After that, cells were imaged under a confocal laser scanning microscopy (CLSM, Olympus Fluoview FV-1000, Tokyo, Japan) to visualize the uptake of DOX and siRNA.

For flow cytometric (FCM) analysis, the two cells were seeded into 6-well culture plates at 1×10^5 cells/well, and treated with free DOX and DOX-loaded BP NSs (DOX equivalent concentration of 5 μg/mL) for 24 h at 37℃. After removing suspension, the cells were washed three times with PBS, digested by trypsin, and finally collected by centrifugation. The cells were observed by a flow cytometer (BD Biosciences, San Jose, CA, USA).

Intracellular siRNA transfection

MCF-7 or MCF-7/ADR cells were seeded at 5 × 10^4 cells per well into 6-well plates and maintained in culture until about 30% confluence. Before transfection, the medium was removed and cells were washed twice with PBS. Then, 2 mL of various complex solutions of siRNA-loaded NSs (0.03 OD siRNA mL^{-1}) were added. Untreated cells were used as a blank control. After 12 h of incubation, the transfection mixture was removed and replaced with fresh culture medium, and maintained for further incubation of 36 h.

Western blot analysis

Samples containing equal amounts of protein from lysates of cultured MCF-7 and MCF-7/ADR cells were analyzed by western blot analysis on a SDS-polyacrylamide gel and transferred to a nitrocellulose membrane by wet transfer. Membranes were blocked in Tris-buffered saline with Tween-20 (TBST) containing 5% nonfat milk powder for 1 h at room temperature and then incubated overnight at 4 °C with 1:5000-diluted primary antibodies. Membranes were then incubated for 1 h at room temperature with 1:5000-diluted species-specific secondary antibodies. The immunoreactive proteins on the membranes were detected using an enhanced chemiluminescence western blotting analysis system. The level of P-gp protein was normalized to the level of GAPDH protein. Semi-quantitative protein estimates were performed by the measure of the band intensity.

For the assessment of P-gp expression in tumors, part of tumor tissues from different groups was added to 1 ml EP tube, followed by the addition of 1 mL RIPA lyses buffer. Then the mixture was transferred to a glass homogenizer and ground in ice water bath for 20 min. Afterwards, the sample in the supernatant was collected by centrifugation at 10000 rpm min^{-1} and mixed up with loading buffer.

***In vitro* phototherapeutic effect**

MCF-7 and MCF-7/ADR cells were seeded in 96-well plates for 24 h. Then the culture medium was replaced with fresh one containing bare BP, BP@PDA-PEG, and BP@PDA-PEG-Apt with different concentrations and further incubated for another 4

h. After that, the cells were treated with NIR irradiation under 808 nm laser (1 Wcm^{-2}, 10 min). After incubation for an additional 12 h, the cell viabilities were evaluated by MTT assay. In brief, 10 μL MTT solution at a concentration of 5 mg mL^{-1} was added to the sample wells and incubated for a 4 h. Subsequently, the mixture was removed and 100 μL DMSO was added to the each well to dissolve the intracellular formazan crystals. The absorbance value was measured at 490 nm using a microplate reader (Bio-Rad Model 680, UK) after gentle shake for 10 min. The percentage of cell viability was measured relative to the media alone group (negative control).

***In vitro* Combined Antitumor Therapy**

Wild-type and MDR MCF-7 cells were seeded on a 96-well plate at a density of 1×10^4 cells/well in 100 μL culture medium. After 24 h, the old cell culture medium was replaced with fresh medium containing different concentrations of DOX or DOX-loaded BP NSs (0.50, 1, 2.5, 5 and 10 μg DOX mL^{-1}) and incubated for 24 h or 48 h. For the NIR irradiation groups, cells were irradiated by NIR laser at 1 W cm^{-2} for 10 min after incubation for 4 h. Then the cell viabilities were evaluated by MTT assay.

Tumor model establishment

Four week-old female sever combined immunodeficient (SCID) mice were used for the animal experiment after purchasing them from the Sun Yat-sen University Laboratory Animal Center. All the protocols for the proposed in vivo experiments were approved by the Administrative Committee on Animal Research in Sun Yat-sen University, with protocol number of IACUC-DD-18-0102. 100 μL of MCF-7/ADR cells suspension in PBS (about 2×10^6 cells) were subcutaneously injected into right flank area of the mice. Tumor sizes were measured with a digital vernier caliper every alternate day. The tumor volume (V) was calculated by the equation: $V = 0.5 \times a \times b^2$, where a and b represented length and width of the tumor, respectively.

Infrared Thermal Imaging

For the photothermal images, MCF-7/ADR tumor-bearing mice were intravenously injected separately with PBS (the control), BP@PDA-PEG and

BP@PDA-PEG-Apt. After 24 h, the mice were anesthetized and the tumor sites were irradiated with a 808nm NIR laser at a power density of 1.5 W cm^{-2} for 5 min. During the irradiation, an infrared thermal image camera was used to monitor the temperature changes and infrared thermographic maps.

Biodistribution analysis

After treatments with DOX，BP-R-D@PDA-PEG and BP-R-D@PDA-PEG-Apt (100 μL, 5 mg mL^{-1}) via tail vein injection, the nude mice were sacrificed at 3 and 24 h post-injection. The major organs including heart, liver, spleen, lung, kidney and tumor tissues of mice were collected. Then the distributions of DOX in various tissues were measured using the Maestro™ Automated *In-Vivo* Imaging system (CRi Maestro™, USA).

Blood pharmacokinetics

Healthy male Sprague–Dawley (SD) rats aged 5–6 weeks were administrated intravenously with DOX, BP-R-D@PDA-PEG and BP-R-D@PDA-PEG-Apt at a DOX single dose of 5 mg kg^{-1} body weight. Blood sample (500 μL) was collected from retro-orbital plexus at different time points and mixed with 3 mL of lysis buffer (1% SDS, 1% Triton-100, 40 × 10^{-3} M Tris acetate, 10 × 10^{-3} M EDTA, and 10 × 10^{-3} M DTT). To extract DOX, 3 mL of HCl/isopropanol was added to the blood. The solution was incubated overnight, and then centrifuged. The supernatant was collected and stored at −20 °C for subsequent analysis. The DOX fluorescence was measured by a microplate reader at an excitation wavelength of 490 nm and an emission wavelength of 580 nm.

***In vivo* tumor therapy**

After the tumor volume reached about 180 mm^3, the tumor-bearing mice were randomly divided into seven groups (5 mice per group) and treated with saline, DOX + siRNA, BP@PDA-PEG-Aptamer, BP-R-D@PDA-PEG, BP-R-D@PDA-PEG-Apt, BP-R-D@PDA-PEG + NIR and BP-R-D@PDA-PEG-Apt + NIR via intravenous injection, respectively. The injection was conducted every 4 days with a DOX dosage of 5 mg kg^{-1} (equivalent DOX dosage for DOX-loaded NPs). The tumor volume and

body weight of the treated-mice were recorded every other day until the end of the treatment. After about 3 weeks of treatment, the nude mice were euthanized. The main organs (heart, liver, spleen, lung, kidney) and tumor tissues were extracted for further studies.

Histological examination

For histological examination, organs including heart, liver, spleen, lung, and kidney were excised, fixed with 10% formalin, embedded with paraffin, sliced into thin sections and stained with hematoxylin and eosin (H&E).

Statistical analysis

Unless stated otherwise, all the experiments were carried out at least three times. The experimental data are expressed as mean ± standard deviation (SD). Statistical analysis was performed by one-way ANOVA followed by Bonferroni test with SPSS 22.0 software. * $P < 0.05$ as statistical significance and ** $P < 0.01$ as extreme statistical significance.

Table S1. The siRNA adsorption capacity of BP NSs under various siRNA concentrations

siRNA equilibrium concentration (nmol mL^{-1})	siRNA EE (%)	siRNA LE (nmol mg^{-1})
2.5	92.1 ± 1.24	2.30 ± 0.019
5.0	92.4 ± 2.55	4.62 ± 0.075
7.5	89.6 ± 1.83	6.72 ± 0.081
10.0	86.5 ± 1.51	8.65 ± 0.088

EE = encapsulation efficiency, LE = loading efficiency, n=3

Table S2. The drug loading capacity of BP@PDA-PEG-Apt under various drug concentrations

DOX concentration (mg mL^{-1})	BP@PDA-PEG-Apt Drug LC (%)
0.4	5.3 ± 0.02
0.6	6.9 ± 0.03
0.8	7.4 ± 0.07
1	8.2 ± 0.11

LC = loading content, n=3

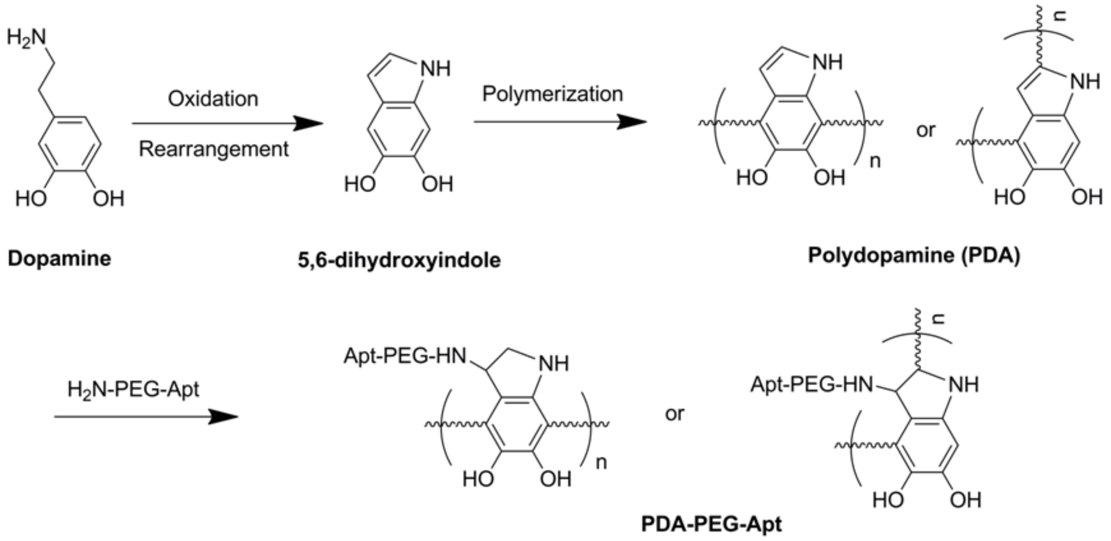

Figure S1. The oxidative self-polymerization mechanism of dopamine and the conjugation mechanism between H_2N-PEG-Apt and PDA coating.

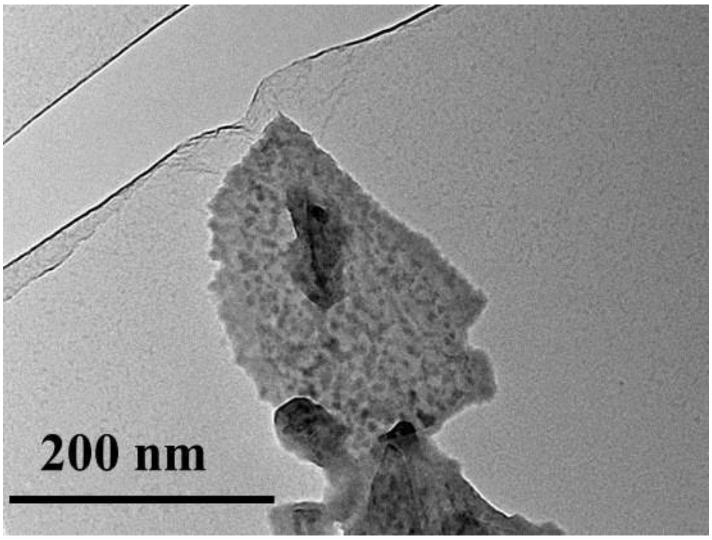

Figure S2. TEM image of BP-R-D.

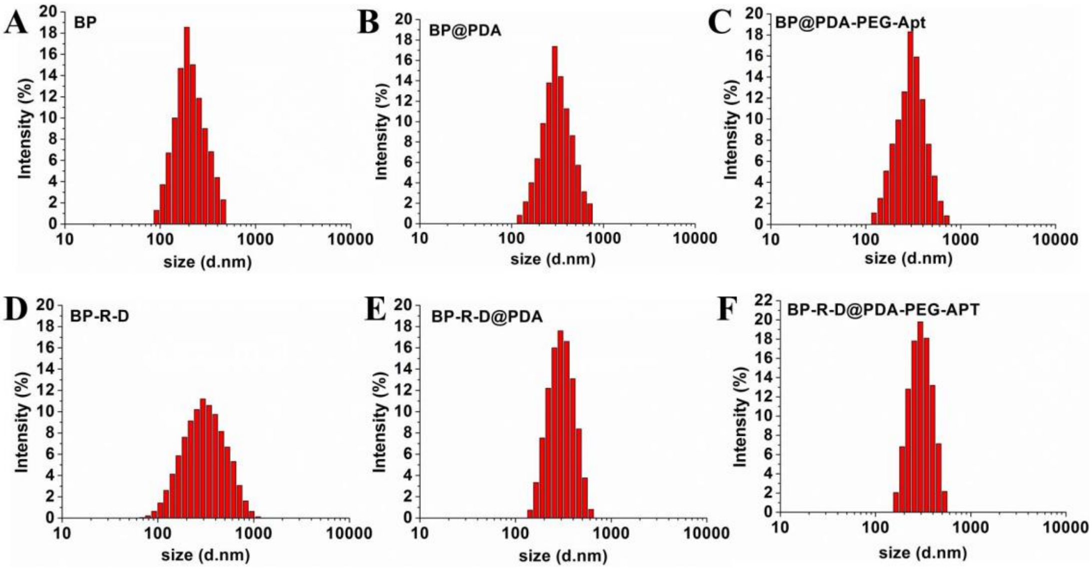

Figure S3. DLS size distribution of different samples.

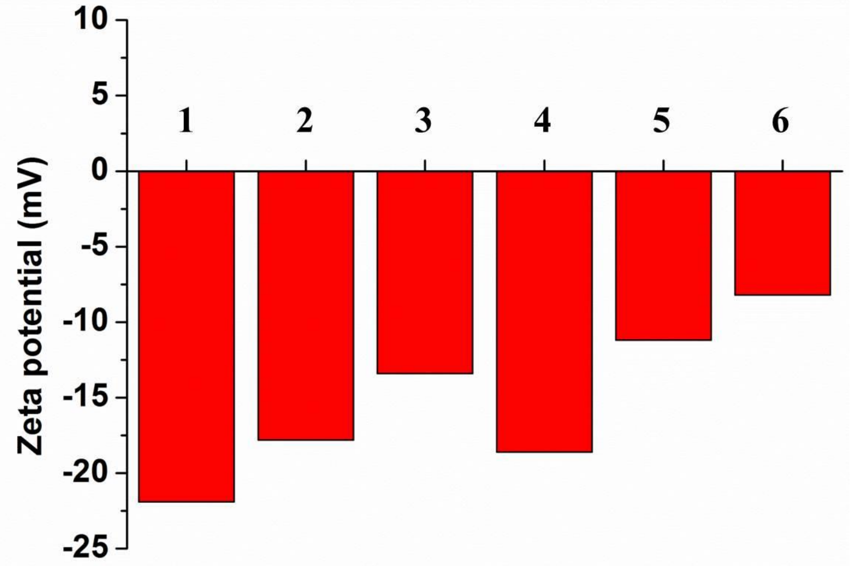

Figure S4. Zeta potentials of BP-based NPs. 1, 2, 3, 4, 5 and 6 represent BP, BP@PDA, BP@PDA-PEG-Apt, BP-R-D, BP-R-D@PDA, and BP-R-D@PDA-PEG-Apt, respectively.

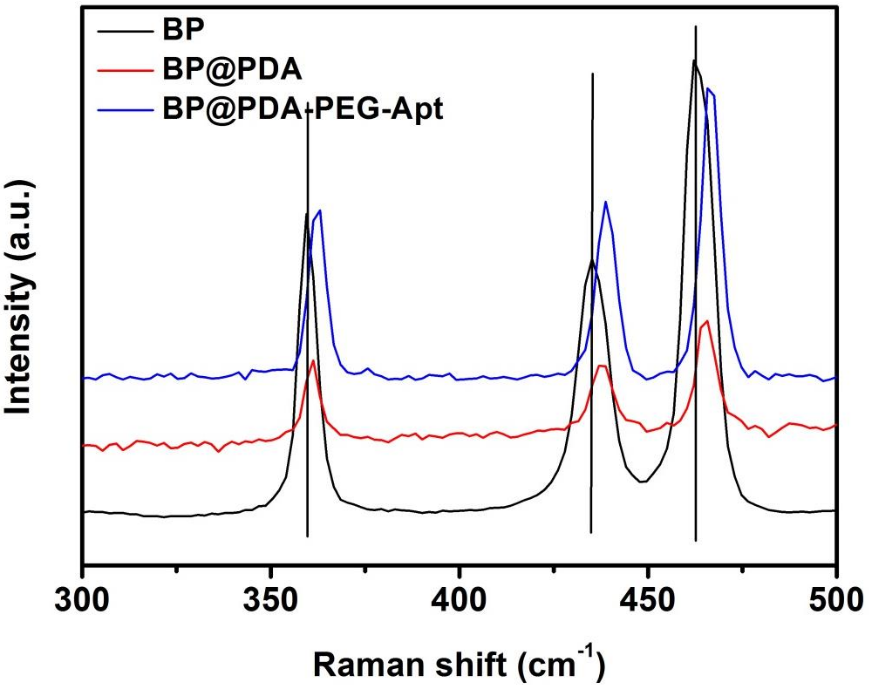

Figure S5. Raman spectra of BP, BP@PDA and BP@PDA-Apt.

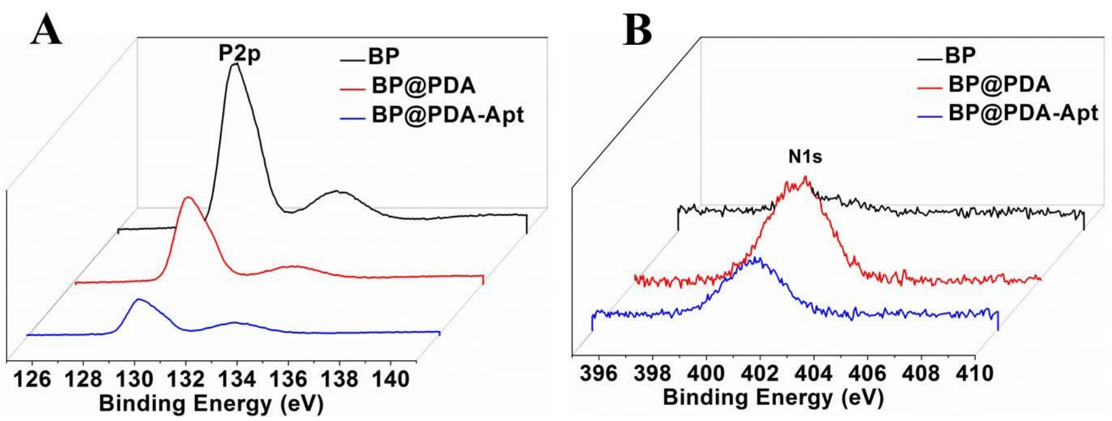

Figure S6. XPS spectra of BP, BP@PDA and BP@PDA-Apt. (A) narrow scan for P2p peaks. (B) narrow scan for N1s peaks.

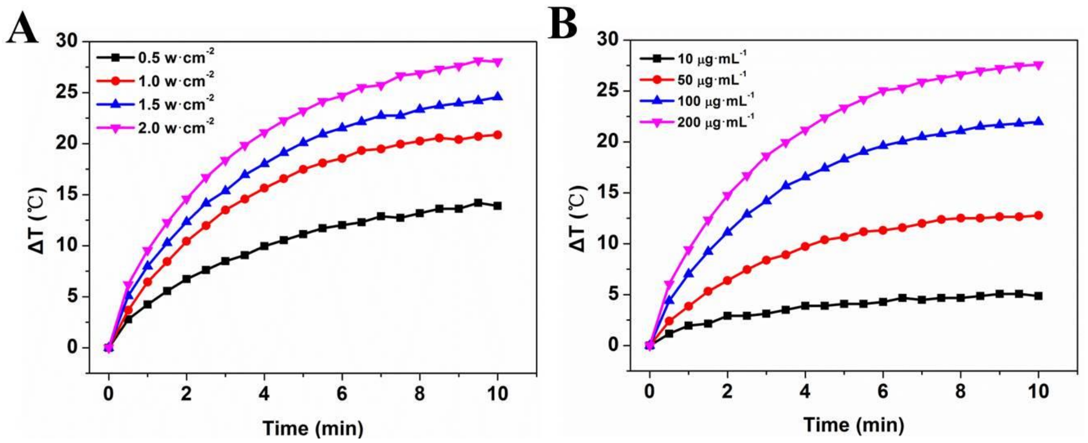

Figure S7. (A) Photothermal heating curves of the BP@PDA-PEG-Apt solution with different concentrations under 808 nm laser irradiation (1.0 W cm^{-2}) for 10 min. (B) Photothermal heating curves of the BP@PDA-PEG-Apt solution under various power intensities.

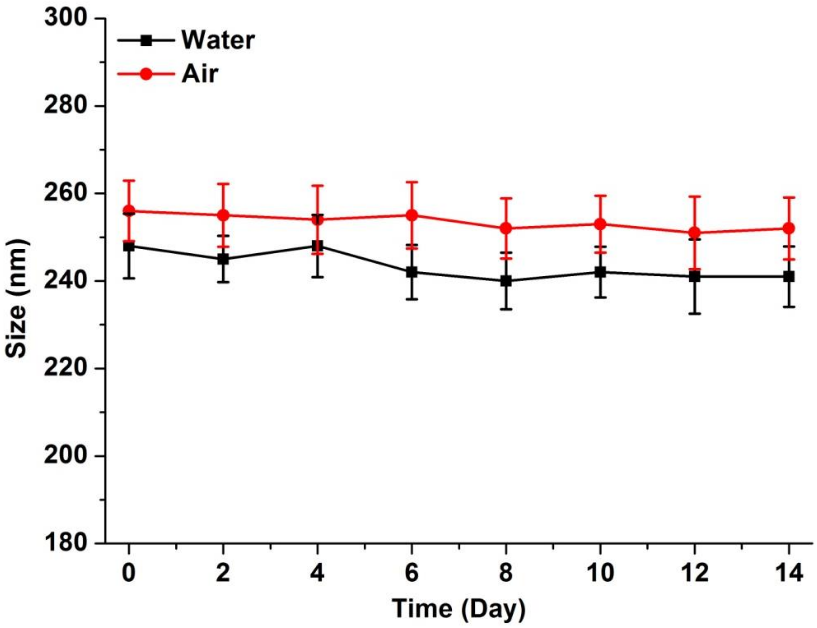

Figure S8. Stability of BP@PDA NSs in water and air, respectively, by monitoring particle size over a span of 14 days.

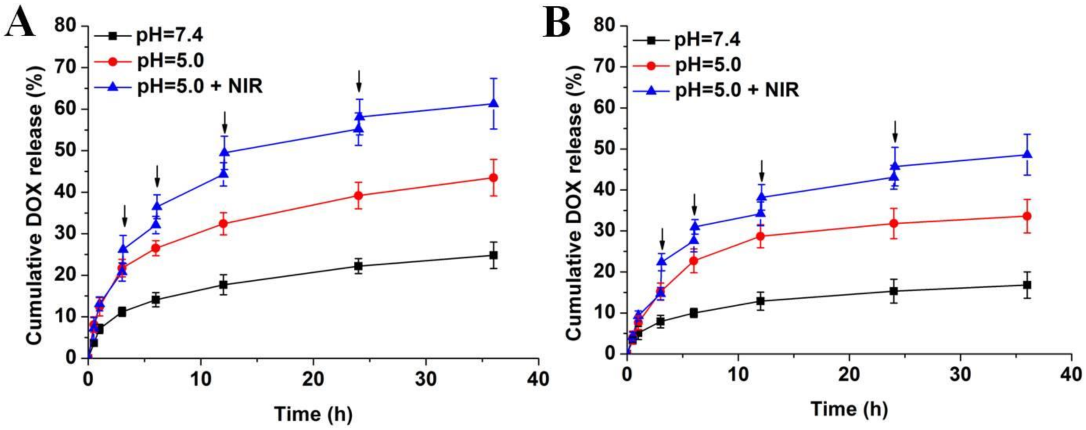

Figure S9. Drug release kinetics at pH = 7.4 and pH = 5.0 (in the absence or presence of 1.0 W cm^{-2} NIR laser), ↓: NIR irradiation for 0.1 h. (A) BP-R-D NSs, and (B) BP-R-D@PDA-PEG-Apt.

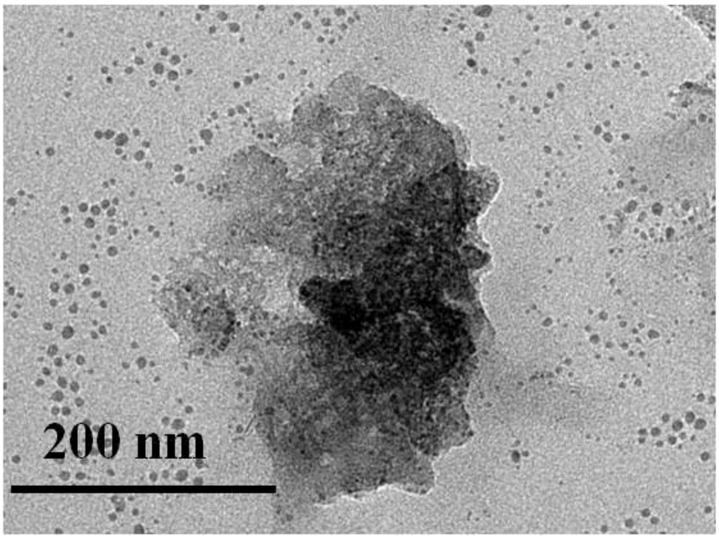

Figure S10. TEM image of BP-D@PDA after NIR irradiation for 30 min.

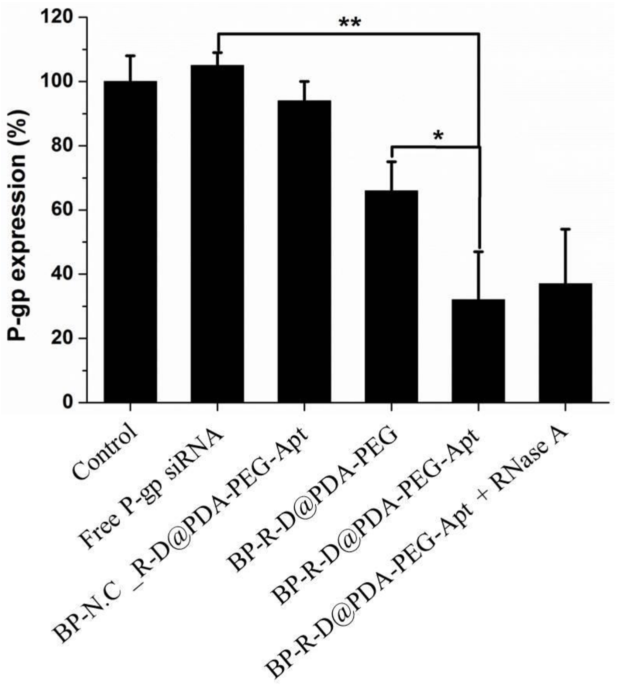

Figure S11. Quantitative analysis of relative P-gp expression (* $P < 0.05$, ** $P < 0.01$).

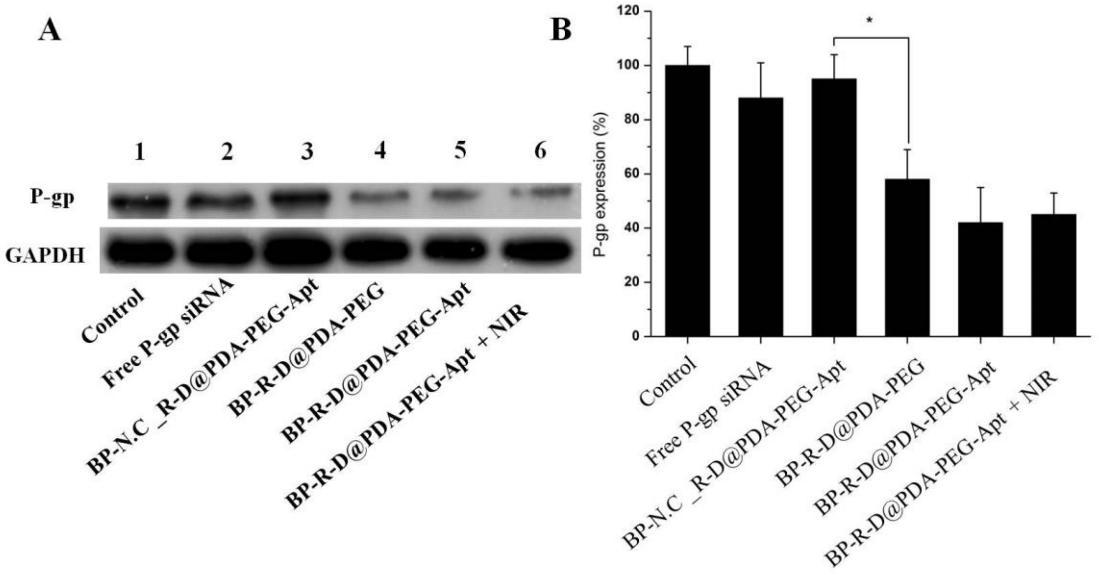

Figure S12. Assessment of P-gp knockdown in the MDR tumors: (A) Western blot analysis. Lane 1: cells without any treatment; Lane 2: free P-gp siRNA; Lane 3: BP-N.C_R-D@PDA-PEG-Apt; Lane 4: BP-R-D@PDA-PEG; Lane 5: BP-R-D@PDA-PEG-Apt; Lane 6: BP-R-D@PDA-PEG-Apt with NIR irradiation. (B) Quantitative analysis of relative P-gp expression (* $P < 0.05$).

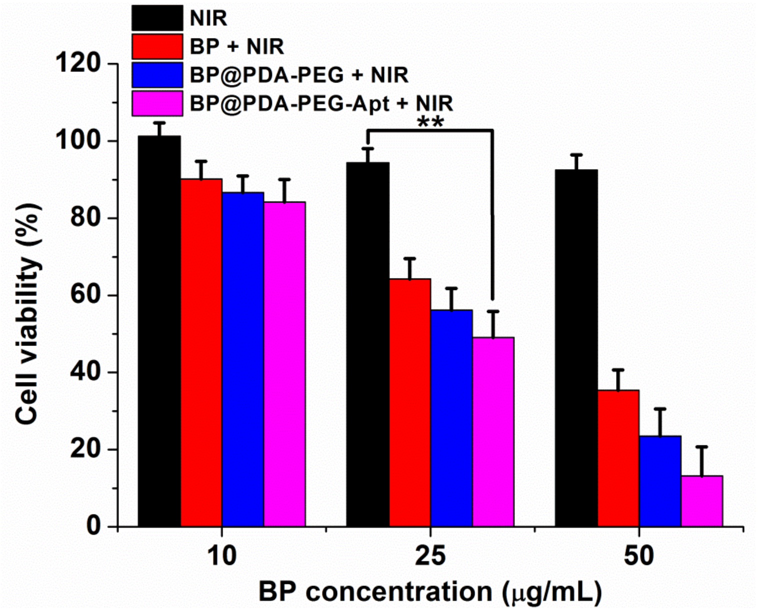

Figure S13. The cell viabilities of MCF-7/ADR cells incubated with various concentrations of BP, BP@PDA-PEG, and BP@PDA-PEG-Apt with NIR laser irradiation (808 nm, 1 W cm^2, 10 min, ** $P < 0.01$).

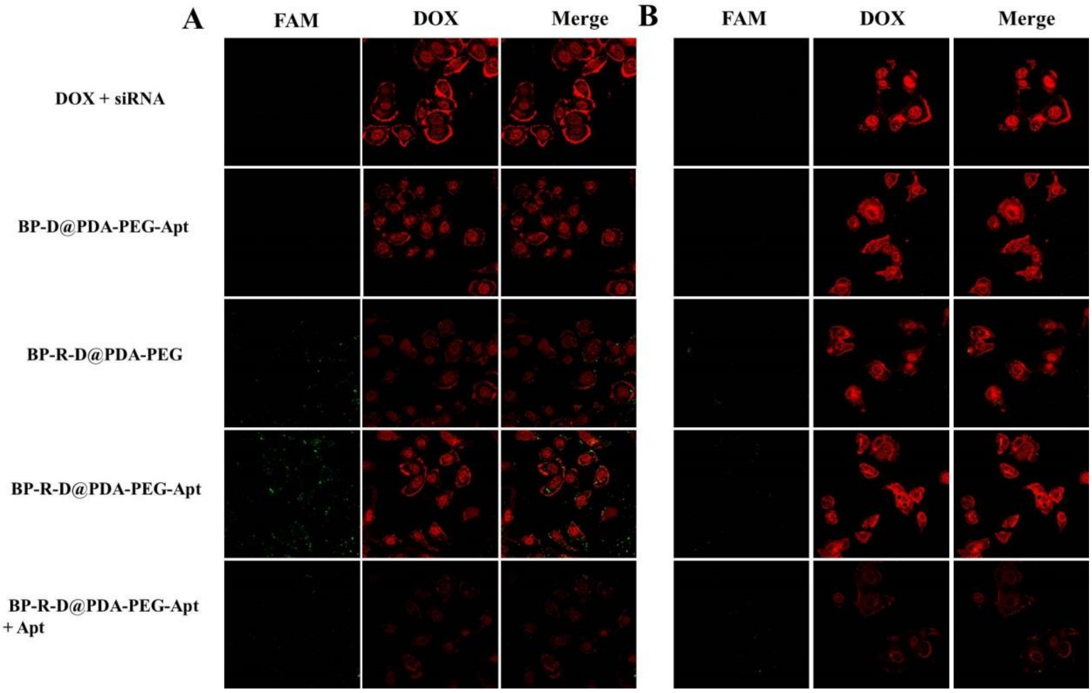

Figure S14. Confocal laser scanning microscopy images of MCF-7 cells after (A) 2 h and (B) 24 h-incubation. Green color: FAM-labeled siRNA. Red color: DOX.

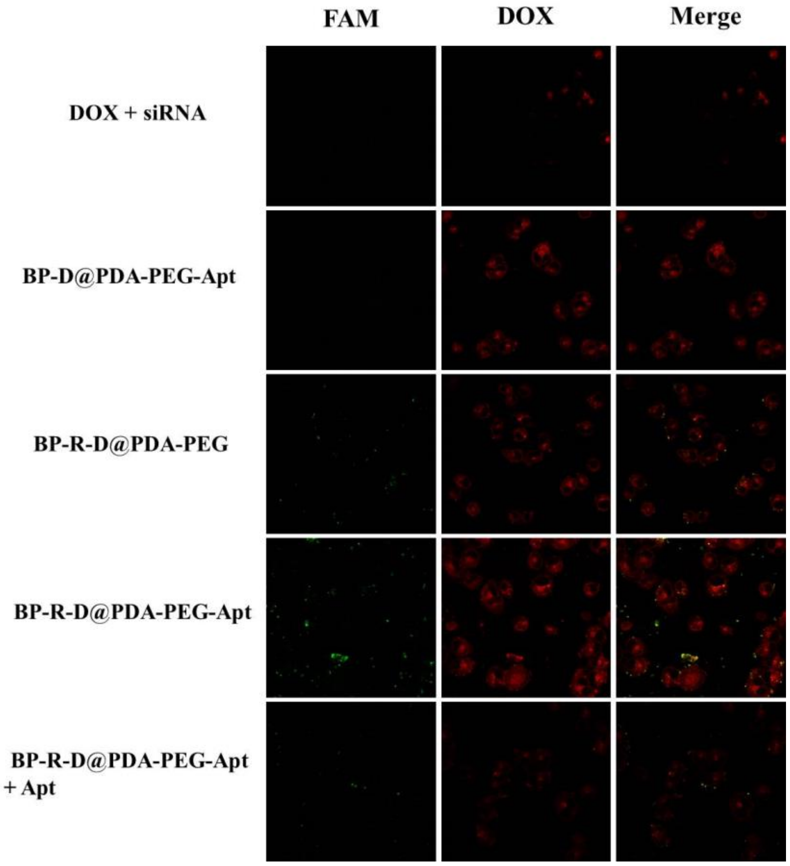

Figure S15. Confocal laser scanning microscopy images of MCF-7/ADR cells after 2 h-incubation.

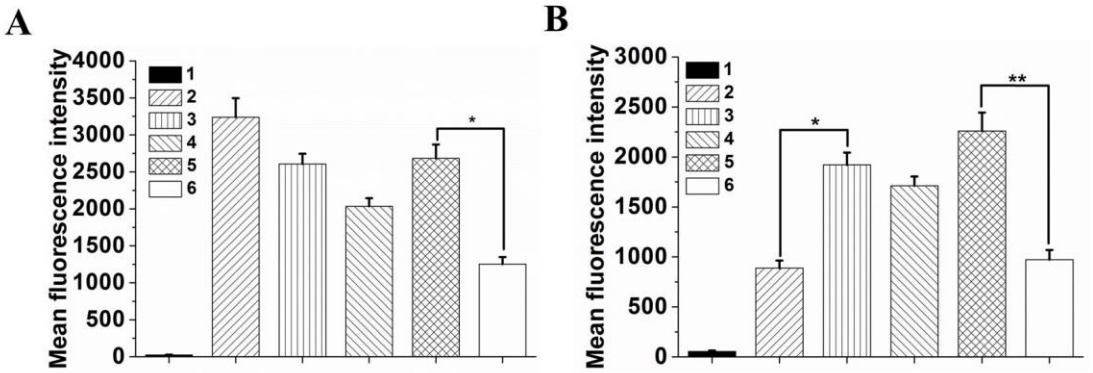

Figure S16. Quantification analysis of DOX fluorescence intensity in (A) MCF-7, and (B) MCF-7/ADR cells after incubation for 26 h. 1, 2, 3, 4, 5 and 6 represent control, free DOX, BP-D@PDA-PEG-Apt, BP-R-D@PDA-PEG, BP-R-D@PDA-PEG-Apt and BP-R-D@PDA-PEG-Apt + free Apt, respectively. (*$P < 0.05$, **$P < 0.01$).

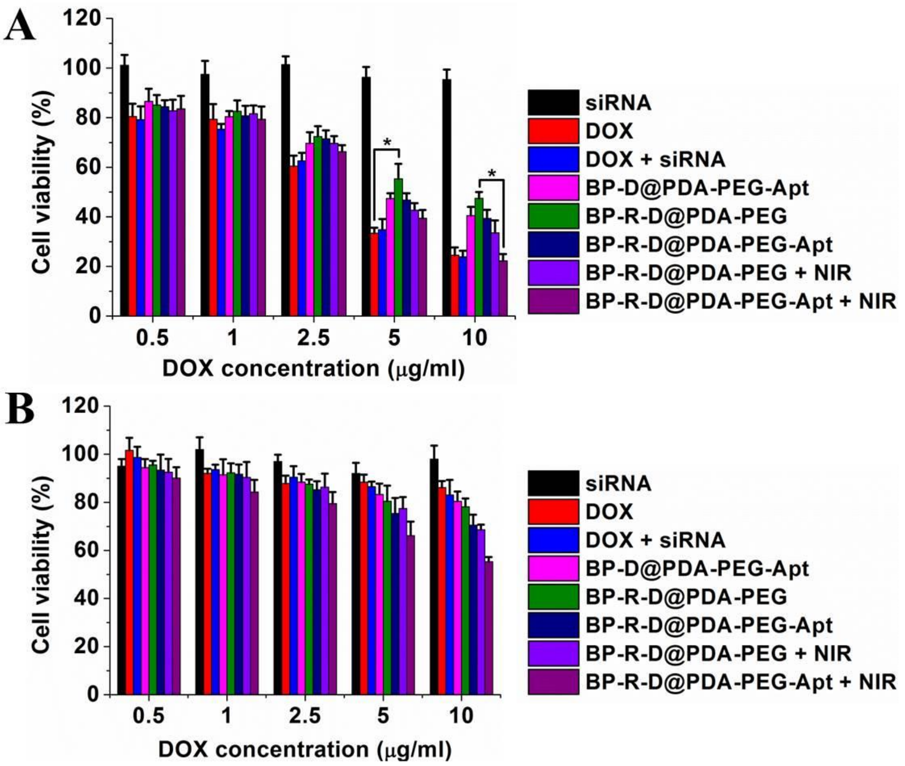

Figure S17. Relative viabilities of tumor cells after different types of treatment for 24 h：(A) MCF-7 cells, and (B) MCF-7/ADR cells (* $P< 0.05$, ** $P< 0.01$).

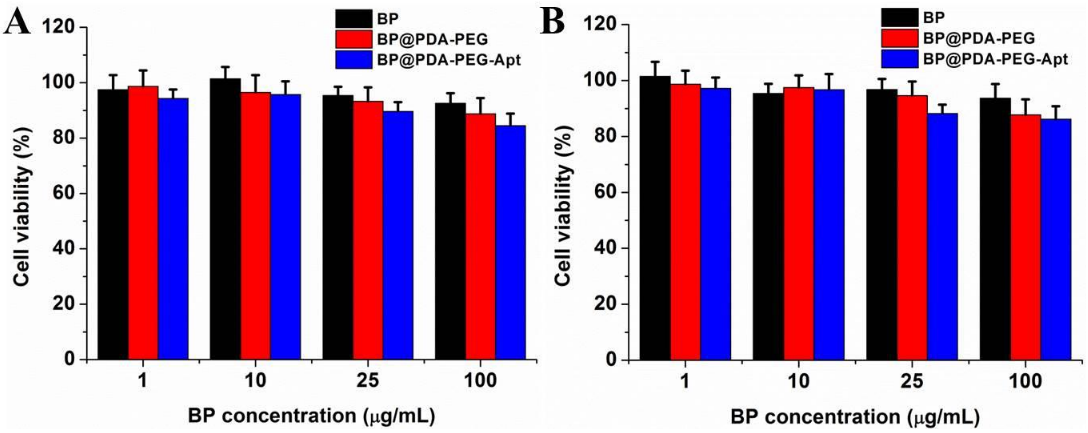

Figure S18. In vitro cytotoxicity of BP, BP@PDA-PEG, and BP@PDA-PEG-Apt test by MTT assay: (A) MCF-7 and (B) MCF-7/ADR.

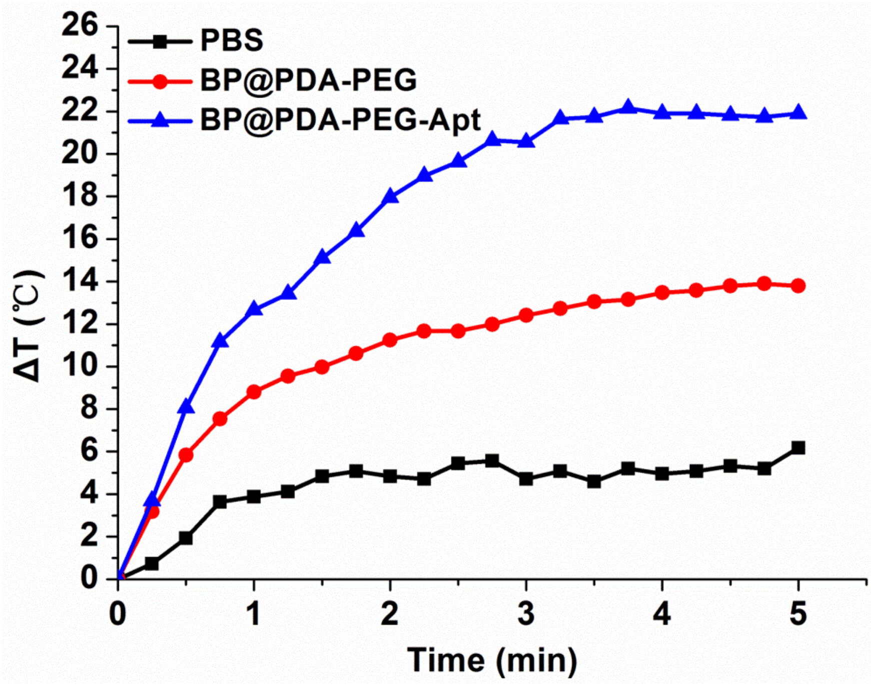

Figure S19. Time-dependent temperature increase of MCF-7/ADR tumor-bearing mice recorded by an IR camera under 808 nm laser (1.5 W cm^{-2}).

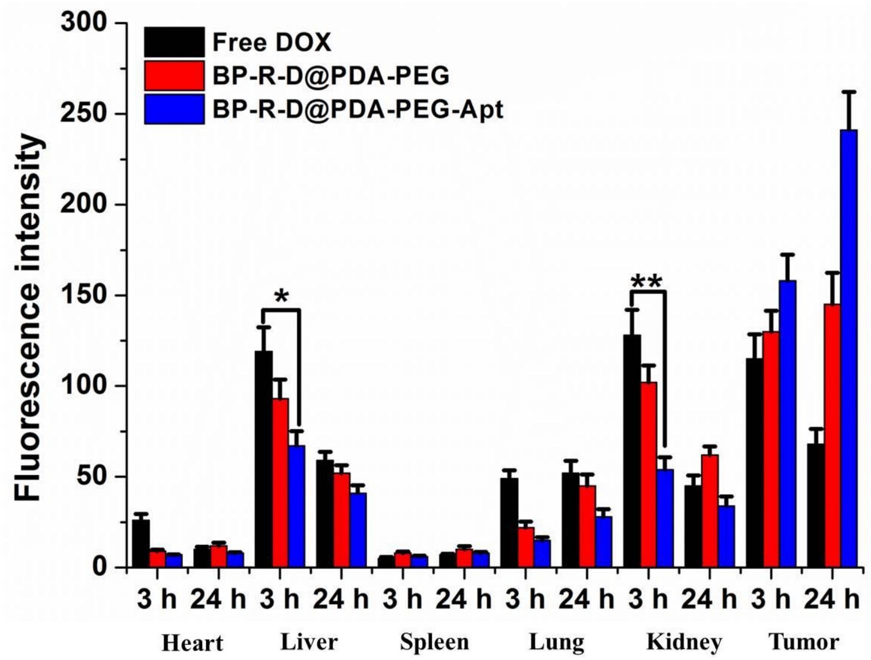

Figure S20. Mean fluorescence intensities of DOX in tumor and major organs (* $P < 0.05$, ** $P < 0.01$).

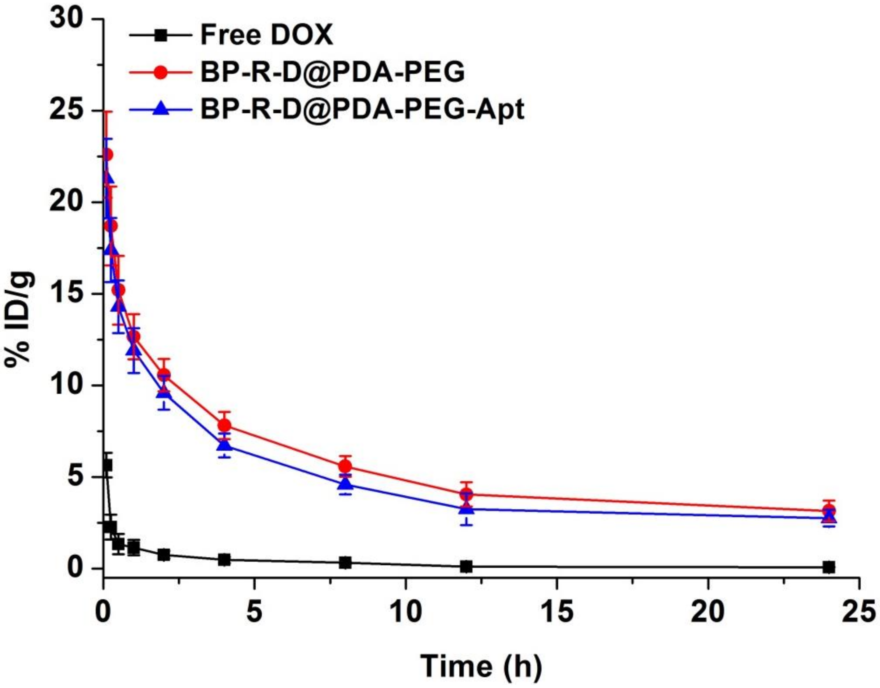

Figure S21. In vivo pharmacokinetics of DOX in blood after Free DOX, BP-R-D@PDA-PEG, and BP-R-D@PDA-PEG-Apt were intravenously injected into the mice through the tail vein at a DOX dose of 5 mg/kg.

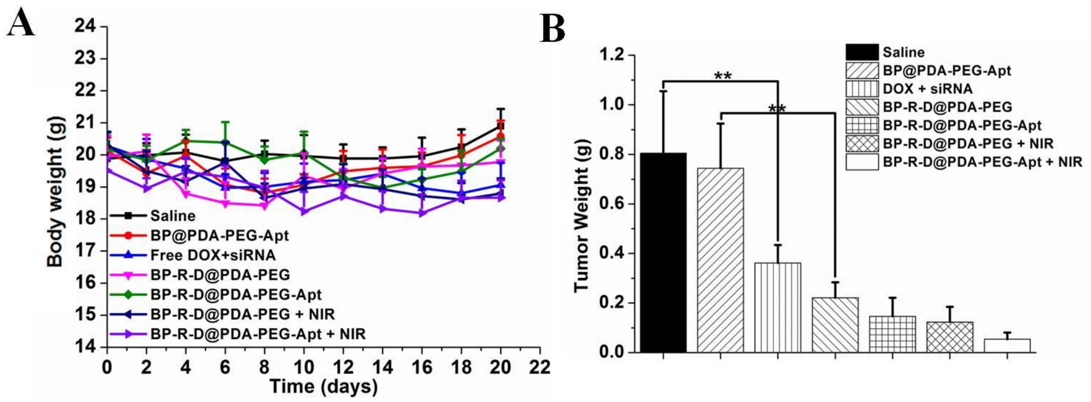

Figure S22. (A) Body weights of mice with various treatments during 20 days evaluation period. (B) Tumor weight of each group taken out from the sacrificed mice at the end point of research (** $P < 0.01$).

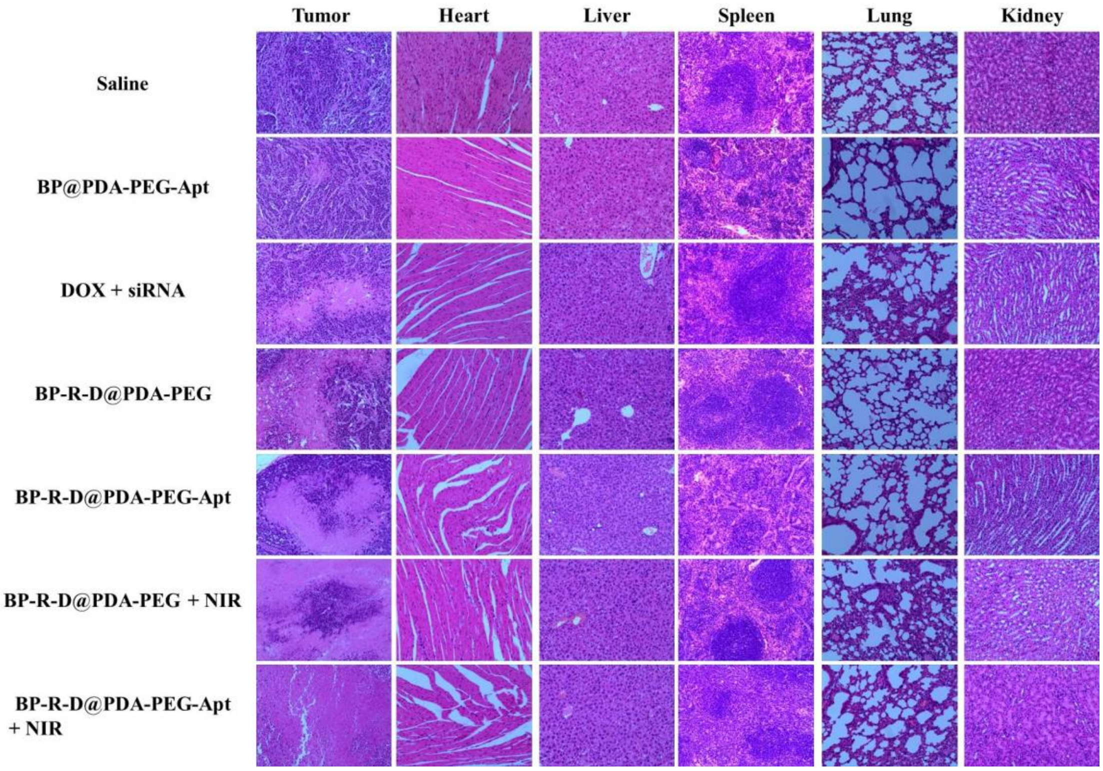

Figure S23. Representative H&E stained images of tumors and major organs including the heart, liver, spleen, lung and kidney collected from MCF-7/ADR tumor-bearing mice after 20 days of combined therapy.

Reference

[1] H. Wang, X. Yang, W. Shao, S. Chen, J. Xie, X. Zhang, J. Wang, Y. Xie, *J. Am. Chem. Soc.* **2015**, 137, 11376.

[2] W. Cheng, C. Liang, X. Wang, H. I. Tsai, G. Liu, Y. Peng, J. Nie, L. Huang, L. Mei, X. Zeng, *Nanoscale* **2017**, 9, 17063.

[3] W. Cheng, C. Liang, L. Xu, G. Liu, N. Gao, W. Tao, L. Luo, Y. Zuo, X. Wang, X. Zhang, X. Zeng, L. Mei, *Small* **2017**, 13.
